# Supplementary material for: In silico design of multi-epitope vaccines against the hantaviruses by integrated structural vaccinology and molecular modeling approaches
Source: PLoS One. 2024 Jul 23;19(7):e0305417. doi: 10.1371/journal.pone.0305417 (PMC11265663; doi:10.1371/journal.pone.0305417)
Supplement: S1 File — (PDF) [file pone.0305417.s001.pdf]

Table 1: Summary of selected CTL epitopes for envelope and nucleoprotein of Hantavirus.

Residue no	Peptide sequence	MHC Binding affinity	Rescale binding affinity	C-terminal cleavage affinity	Transport affinity	Prediction score	MHC-I Binding	Antigenicity score (0.4)
**Envelope**								
**139**	QTHCQPTVY	0.50	2.13	0.94	3.08	2.42	Yes	1.04
**531**	ILEKVKIEY	0.31	1.33	0.97	2.71	1.61	Yes	0.92
**32**	HTVGLGQGY	0.29	1.26	0.80	2.74	1.52	Yes	0.69
**Nucleoprotein**								
**90**	LKEKSSLRY	0.25	1.10	0.92	2.81	1.37	Yes	0.70
**257**	FMSTNKMYF	0.16	0.69	0.77	2.71	0.94	Yes	0.56
**353**	KLKKKSAFY	0.11	0.49	0.96	2.88	0.77	Yes	1.27
**Proteome**								
**139**	QTHCQPTVY	0.50	2.13	0.94	3.08	2.42	Yes	1.04
**531**	ILEKVKIEY	0.31	1.33	0.97	2.71	1.61	Yes	0.92
**32**	HTVGLGQGY	0.29	1.26	0.80	2.74	1.52	Yes	0.69
**90**	LKEKSSLRY	0.25	1.10	0.92	2.81	1.37	Yes	0.70
**257**	FMSTNKMYF	0.16	0.69	0.77	2.71	0.94	Yes	0.56
**353**	KLKKKSAFY	0.11	0.49	0.96	2.88	0.77	Yes	1.27

Table 2: Summary of Helper T-cell epitopes selected for envelope and nucleoprotein of Hantavirus using the IEDB MHC-II module

Sr. No.	Allele	Method	Peptides	Percentile Rank	Antigenicity score (0.4)
Envelope					
1	HLA-DRB4*01:01	Consensus	VVLVVILILSIIMFS	0.02	0.59
2	HLA-DRB4*01:01	Consensus	VVVLVVILILSIIMF	0.02	0.50
3	HLA-DRB4*01:01	Consensus	LVVILILSIIMFSVL	0.08	0.53
4	HLA-DRB4*01:01	Consensus	VLVVILILSIIMFSV	0.09	0.54
Nucleoprotein					
1	HLA-DRB1*03:01	Consensus	MGIQLDQKIIILYML	0.05	0.76
2	HLA-DRB1*03:01	Consensus	QSMGIQLDQKIIILY	0.05	0.76
3	HLA-DRB1*03:01	Consensus	SMGIQLDQKIIILYM	0.05	0.84
4	HLA-DRB1*03:01	Consensus	TQSMGIQLDQKIIIL	0.06	0.73
Proteome					
1	HLA-DRB4*01:01	Consensus	VVLVVILILSIIMFS	0.02	0.59
2	HLA-DRB4*01:01	Consensus	LVVILILSIIMFSVL	0.08	0.53
3	HLA-DRB4*01:01	Consensus	VLVVILILSIIMFSV	0.09	0.54
4	HLA-DRB1*03:01	Consensus	MGIQLDQKIIILYML	0.05	0.76
5	HLA-DRB1*03:01	Consensus	QSMGIQLDQKIIILY	0.05	0.76
6	HLA-DRB1*03:01	Consensus	SMGIQLDQKIIILYM	0.05	0.84

Table 3: Results of ABCPred: Summary of linear B cell epitopes.

Sr. No.	Epitope	Position	Score	Antigenicity score (0.4)
Envelope				
1	LIILKCLRVLTFSCSHYTNE	506	0.96	0.62
2	KTDLELDFSLPSSSSYSYRR	673	0.90	0.95
Nucleoprotein				
1	KDAEKAVEVDPDDVNKSTLQ	26	0.91	0.61
2	FPAQVKARNIISPVMGVIGF	206	0.90	0.99
Proteome				
1	LIILKCLRVLTFSCSHYTNE	506	0.96	0.62
2	KTDLELDFSLPSSSSYSYRR	673	0.90	0.95
3	KDAEKAVEVDPDDVNKSTLQ	26	0.91	0.61
4	FPAQVKARNIISPVMGVIGF	206	0.90	0.99

Table 4: The physiochemical properties of constructed vaccines.

Vaccine construct	Molecular weight (kDa)	Theoretical PI	Half-life in E. Coli	Instability index	Aliphatic index	GRAVY
**Env-Vac**	24418.68	9.23	>10 hours	42.62	127.96	0.902
**NP-Vac**	24681.58	9.57	>10 hours	44.55	94.96	0.150
**Com-Vac**	37852.45	9.51	>10 hours	44.81	109.02	0.410

Table 6: Summary of binding free energies for docked complexes (Multi-Epitopes Vaccine constructs-TLR3).

Complexes Names	VDW	ELE	GB	SA	TOTAL
**Env-Vac-TLR3**	-168.66	-924.98	1064.64	-21.01	-50.02
**NP-Vac-TLR3**	-221.99	-1241.91	1469.99	-30.23	-24.13
**Com-Vac-TLR3**	-149.75	-911.38	1017.53	-18.69	-62.30
